# Supplementary material for: Assessment of protein–protein interfaces in cryo-EM derived assemblies
Source: Nat Commun. 2021 Jun 7;12:3399. doi: 10.1038/s41467-021-23692-x (PMC8184972; doi:10.1038/s41467-021-23692-x)

Assessment of protein-protein interfaces in cryo-EM derived assemblies

Sony Malhotra[12]*, Agnel Praveen Joseph[2], Jeyan Thiyagalingam[2], Maya Topf[1,3]*

[1]Institute of Structural and Molecular Biology, Department of Biological Sciences, Birkbeck College, University of London, Malet Street, London WC1E 7HX, United Kingdom

[2]Scientific Computing Department, Science and Technology Facilities Council, Didcot OX11 0FA, United Kingdom

[3]Centre for Structural Systems Biology, Leibniz-Institut für Experimentelle Virologie and Universitätsklinikum Hamburg-Eppendorf (UKE), 22607 Hamburg, Germany

*Co-corresponding authors

Email: sony.malhotra@stfc.ac.uk (SM), m.topf@cryst.bbk.ac.uk (MT)

Supplementary Table 1: Oligomeric scores for negatively-scoring models using SVM-based model for two of CASP13 cryo-EM targets: T1020o and T0995o. Different scores which assess the oligomeric quality are listed for the models that scored negative using our interface-based SVM score. Interface contact similarity (F1) and interface patch scores (Jaccard coefficient) range from 0 (worst) to 1(best). GDTo and lDDT(oligo) (local distance difference test) consider the whole oligomeric assembly and range from 0 (different quaternary structure) to 1(similar quaternary structure) and are computed after chain mapping combinations between the target to those of the model using QS algorithm (see Methods).

**Target**	**Model**	**GDTo**	**lDDT(oligo)**	**Jaccard coefficient**	**F1**
**T1020o**	TS114_4o	0.2638	0.553	0.16	0
	TS008_4o	0.2212	0.529	0.09	0
	TS208_5o	0.274	0.568	0.14	0
	TS208_3o	0.2721	0.57	0.13	0
	TS208_4o	0.2742	0.577	0.12	0
	TS208_1o	0.2734	0.567	0.12	0
	TS432_3o	0.0482	0.166	0.03	0
	TS047_1o	0.0338	0.176	0	0
	TS135_3o	0.2117	0.507	0.15	0
**T0995o**	TS008_5o	0.3433	0.513	0.39	0.274
	TS008_4o	0.2205	0.506	0.36	0.24
	TS329_1o	0.2864	0.564	0.38	0.167
	TS397_3o	0.2368	0.26	0.48	0.244
	TS397_4o	0.3669	0.226	0.4	0.166
	TS397_2o	0.3669	0.216	0.39	0.1
	TS470_3o	0.1029	0.454	0.09	0.037
	TS397_1o	0.2382	0.258	0.3	0.069
	TS460_1o	0.104	0.59	0.24	0.022
	TS196_3o	0.1081	0.579	0.1	0
	TS117_1o	0.103	0.559	0.16	0
	TS114_5o	0.0923	0.323	0.13	0.004

Supplementary Table 2: Density correlation score (CCC) and local score (SMOC) averaged over interface residues for the interfaces in target structure (T1020o) and model (TS208_1o) at target map resolution of 3.3 Å and low-pass filter resolutions (5, 8, 10 and 12Å). For the model TS208_1o, SMOC was not calculated for interfaces formed by chain B and C (marked as NA) as the number of the residues at the interface was lower than the cutoff.

Resolution (Å)	Target (T1020o)			TS208_1o		
	Chains Target	CCC (Chimera)	SMOC	Chains Model	CCC (Chimera)	SMOC
3.3	BC	0.77	0.69	AB	0.40	NA
	BA	0.77	0.69	AC	0.39	0.27
	AC	0.77	0.68	CB	0.24	0.22
5	BC	0.77	0.72	AB	0.52	NA
	BA	0.77	0.73	AC	0.50	0.39
	AC	0.77	0.72	CB	0.38	0.32
8	BC	0.79	0.73	AB	0.68	NA
	BA	0.79	0.73	AC	0.65	0.53
	AC	0.79	0.72	CB	0.58	0.53
10	BC	0.85	0.78	AB	0.79	NA
	BA	0.85	0.77	AC	0.76	0.63
	AC	0.86	0.77	CB	0.71	0.67
12	BC	0.88	0.85	AB	0.85	NA
	BA	0.88	0.85	AC	0.83	0.74
	AC	0.88	0.85	CB	0.80	0.77

Supplementary Table 3: Density correlation score (CCC) and local score (SMOC) averaged over interface residues for the interfaces in the target structure (T0002) and model (TS164_1) at target map resolution of 3.3 Å and low-pass filter resolutions (5, 8 and 10Å).

Resolution (Å)	Alpha-ring interface				Beta-ring interface			
	CCC (Chimera)		Local Score (SMOC)		CCC (Chimera)		Local Score (SMOC)	
	Target P_Q	Model F_C	Target P_Q	Model F_C	Target X_Y	Model n_d	Target X_Y	Model n_d
3.3	0.85	0.73	0.78	0.23	0.85	0.65	0.79	0.26
5	0.89	0.83	0.84	0.39	0.89	0.73	0.83	0.51
8	0.93	0.91	0.93	0.57	0.93	0.83	0.93	0.71
10	0.94	0.92	0.95	0.67	0.95	0.89	0.96	0.80

Supplementary Table 4: Interface assessment for fitted models in the EM DataBank. The fitted models and crystal structure IDs with their chains identifier forming the equivalent interfaces are listed along with the interface RMSD (iRMSD), fraction of native interface residues aligned (f_{Nal}) and the PI-score using our classifier.

Fitted model	Crystal structure	Chains of fitted model at interface	Chains of crystal structure at interface	iRMSD (Å), f_{Nal}	PI-score for fitted model
3J2W	3N44	NB	BF	2.08, 0.34	-1.67
6MZD	6F3T	LF	FA	1.68, 0.4	-1.99

Supplementary Figure 1: Scoring the interfaces in the oligomeric target T0995o (CASP13).
The chains from the labelled structures are marked appropriately and the PI-score is marked for the interface.
a. The target structure is shown in a golden rod fitted in the cryo-EM density. The dimer interface is scored positive.
b. Model TS008_2o, which is the best scoring model in terms of CCC, is superposed onto the target structure. The model is scored positive for the dimer interface.
c and d. Model TS117_1o and TS008_5o, respectively, superposed onto the target structure, which is scored negative.

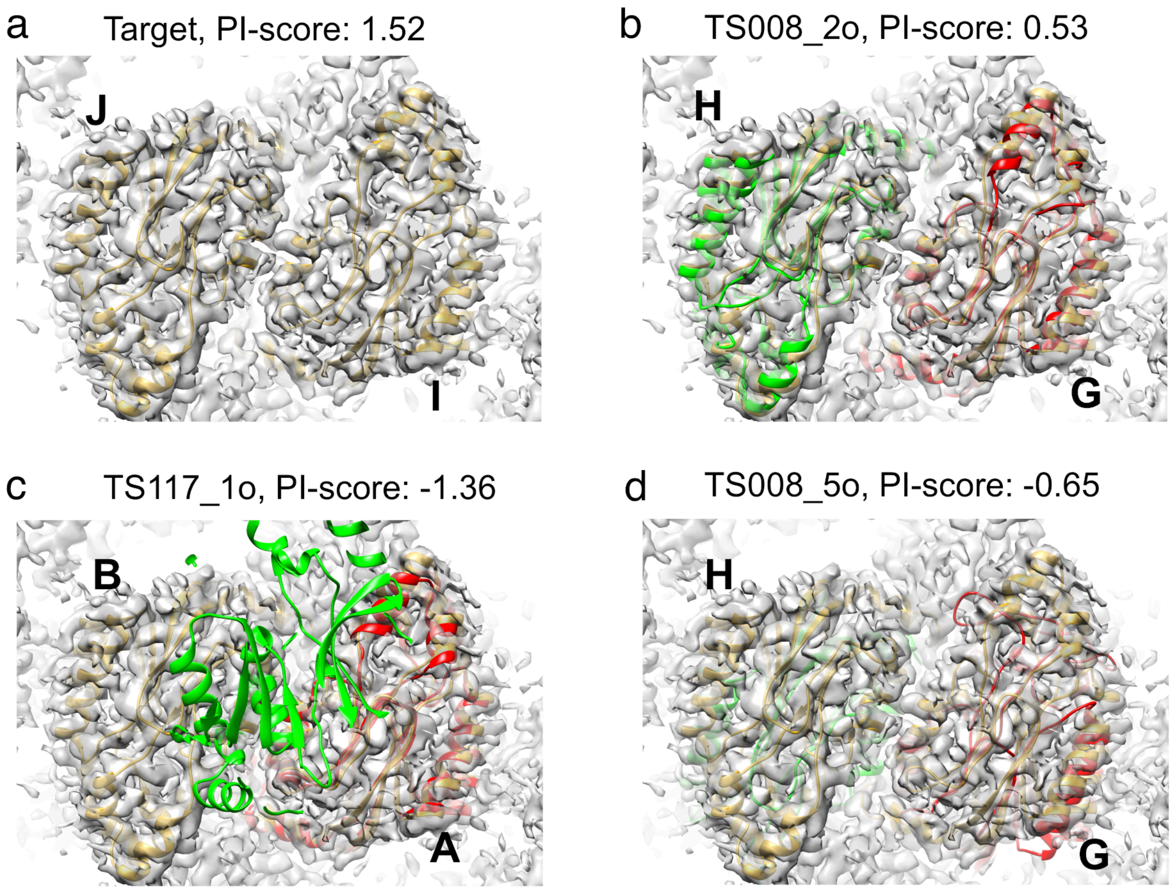

Supplementary Figure 2: Interface between the small subunits (S5-S28) in the nsp1-40S ribosome bound structure (EMD-11301, 3Å).
a. nsp1 (blue) bound human 40S ribosome structure, where small subunits S5 and S28 are shown in green and red respectively.
b. Close-up of the interface between S5-S28. Interface residues are shown as grey sticks. Steric clashes were observed at the interface and are highlighted in spheres.
c. S5(cyan)-S28(pink) upon refinement in Coot. The residues pairs at the interface which were re-modeled are shown as spheres.

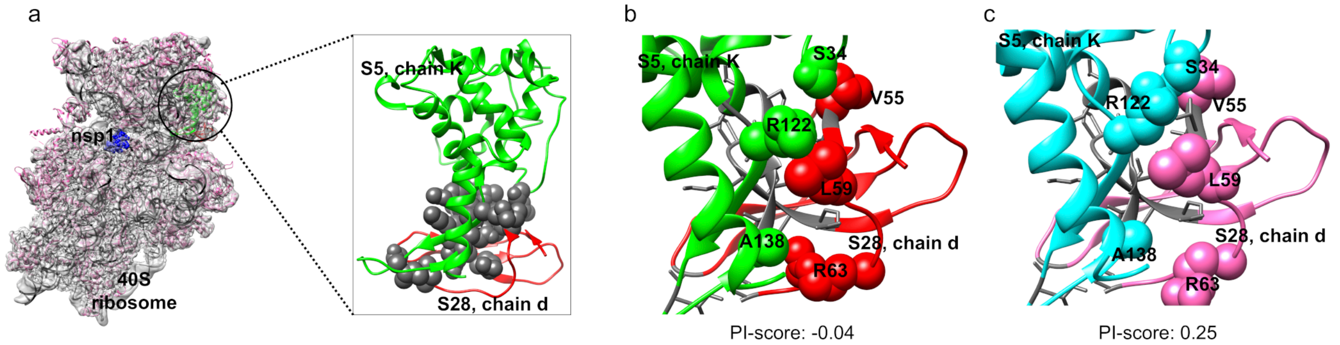

Supplementary Figure 3: Comparison of machine learning based score vs protein-protein interface statistical potentials. On the X-axis is the combined machine learning statistical potential score (ML_stat_pot combined). The high-resolution complexes (PD1) are shown in yellow; the native-like complexes (PD2) are in green and the negative dataset complexes are shown in skyblue. w1 and w2 are the weights assigned to the machine learning based score and the statistical potentials respectively.

a. Performance of two scores are shown for PD2 and ND (negative dataset, with interfaces far from native derived using docking)

b. Performance of two scores for the dataset including native complexes (positive dataset: PD1+PD2 and negative dataset: ND).

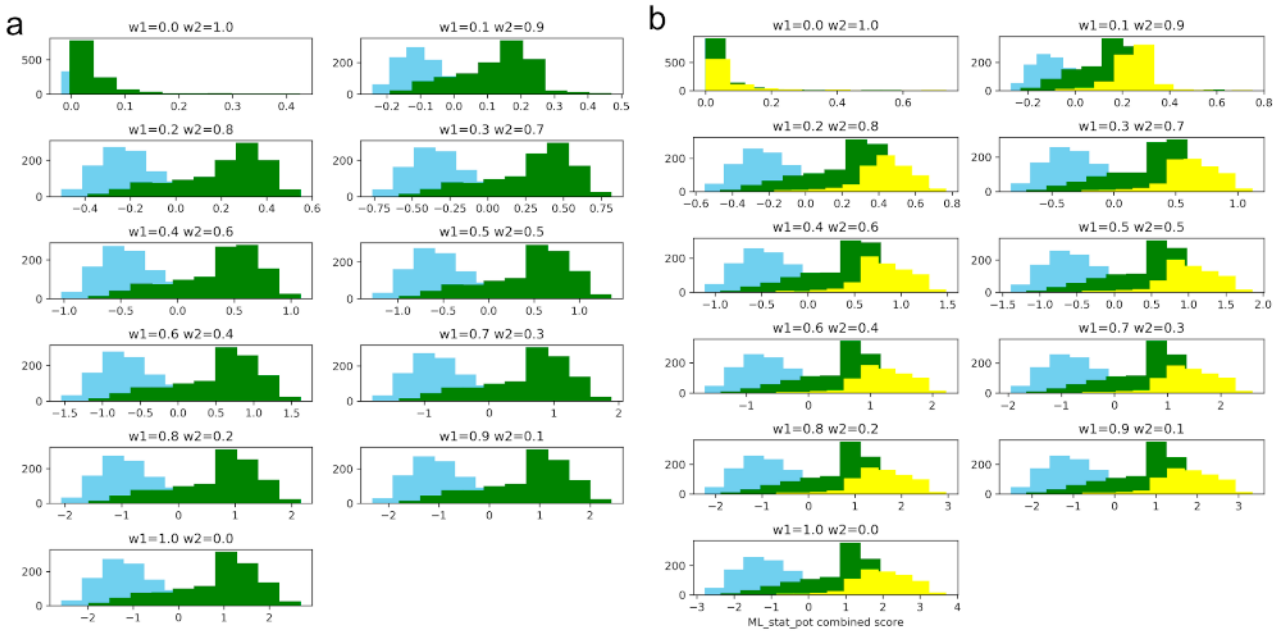

Supplementary Figure 4: Performance of the weighted combined score for the submitted pool of models for the EM-model challenge targets, T0002 and T0003. a, d. On the X-axis is the interface RMSD (iRMSD) between the model and the equivalent target interface. The combined score is shown in green, iCCC is in cyan and PI-score is in orange.
a. The combined score for the interface NM for the model EM133_1 (T0002) is highlighted with a black circle.
b. Chains M (green) and N (red) of model EM133_1 in map (EMD-5623) superposed on the target structure (PDB ID: 3j9i),
c. Chains M and N of the model EM133_1, colored according to the local CCC score (see Methods).
d. The combined score for the interface EF for the model EM164_1 (T0003) is highlighted with black circle.
e. Chains E (red) and F (green) of model EM164_1 in map (EMD-6422) superposed on the target structure (PDB ID: 1ss8).
f. Chains M and N of model EM164_1, colored according to the local CCC score (see Methods).

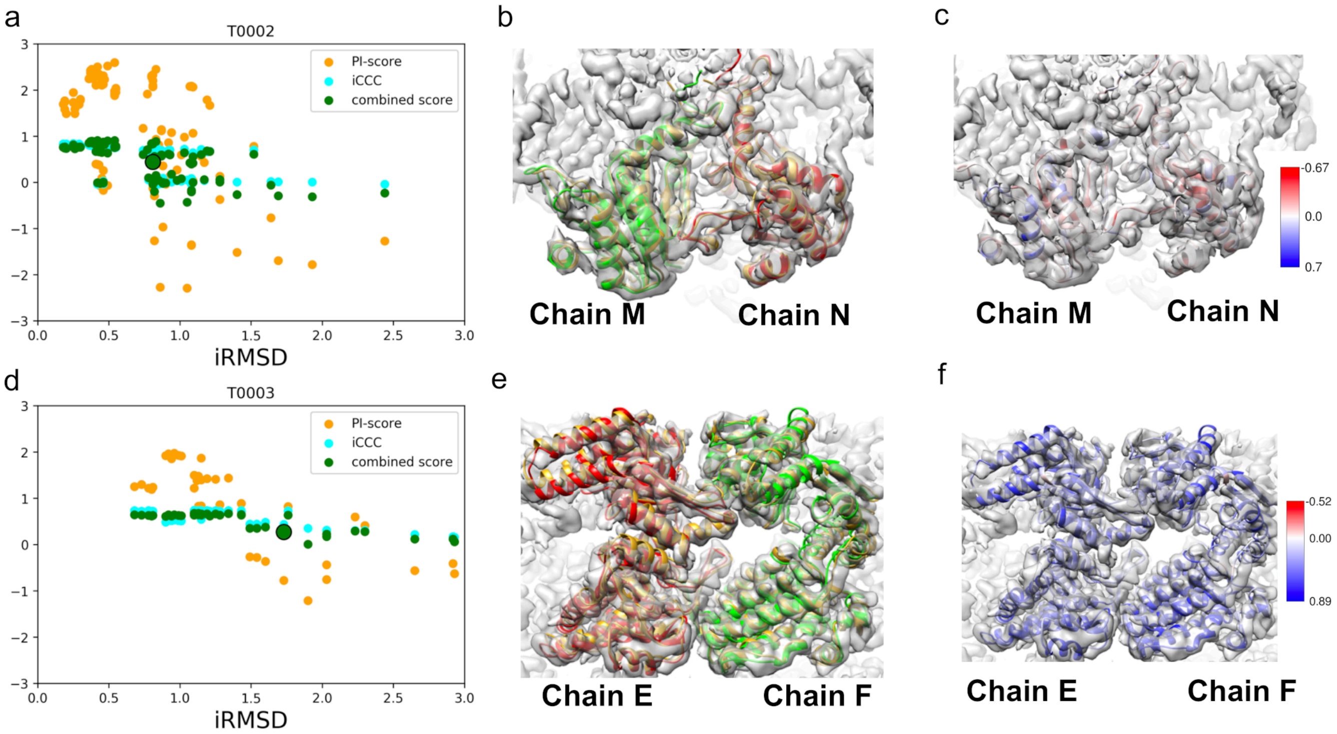

Supplement: Supplementary file 1 — Supplementary Information [file 41467_2021_23692_MOESM1_ESM.pdf]
