# Supplementary material for: Assessment of protein–protein interfaces in cryo-EM derived assemblies
Source: Nat Commun. 2021 Jun 7;12:3399. doi: 10.1038/s41467-021-23692-x (PMC8184972; doi:10.1038/s41467-021-23692-x)
Supplement: Supplementary file 2 — Description of Additional Supplementary Files [file 41467_2021_23692_MOESM2_ESM.pdf]

Description of Additional Supplementary Files

Supplementary Data 1: Assessment scores for interfaces in the models submitted for three of the CASP13 cryo-EM targets (T0984o, T1020o and T0995o).

Supplementary Data 2: Assessment scores for interfaces in the models scored for the EM model challenge targets.

Supplementary Data 3: Assessment scores for interfaces in the fitted models obtained from EMDB at resolution better than 4Å.

Supplementary Data 4: Assessment scores for interfaces in the fitted models obtained from EMDB at resolution range 4-8 Å.

Supplementary Data 5: Assessment scores for interfaces in the fitted models obtained from EMDB at resolution range 8-12 Å.

Supplementary Data 6: Assessment scores for interfaces in the fitted models for SARS-CoV-2 obtained from EMDB.
